# Higher Maternal Protectiveness Is Associated with Higher Odds of Child Overweight and Obesity: A Longitudinal Australian Study

**DOI:** 10.1371/journal.pone.0100686

**Published:** 2014-06-23

**Authors:** Kirsten J. Hancock, David Lawrence, Stephen R. Zubrick

**Affiliations:** Telethon Kids Institute, The University of Western Australia, Perth, Western Australia, Australia; Geisel School of Medicine at Dartmouth College, United States of America

## Abstract

In recent years there has been an increasing interest in overprotective parenting and the potential role it plays in child development. While some have argued that a trend towards increased parental fear and reduced opportunity for independent mobility may be linked to increasing rates of child overweight and obesity, there is limited empirical information available to support this claim. Using data from the *Longitudinal Study of Australian Children*, this study aimed to examine the longitudinal relationships between maternal protectiveness and child overweight and obesity. A cohort of 4–5 year old children was followed up at 6–7, 8–9 and 10–11 years of age (n  =  2596). Measures included a protective parenting scale administered when children were 6–7 and 8–9 years of age, child body mass index (BMI), family characteristics including household income, neighbourhood disadvantage, child's position amongst siblings, and maternal BMI, education, employment, mental health and age at first birth. International Obesity Taskforce age- and sex-specific BMI cut points were used to determine if children were in the normal, overweight or obese BMI range. There was no association between maternal protectiveness and the odds of children being overweight or obese at age 4–5, 6–7 or 8–9 years. However at age 10–11 years, a 1 standard deviation increase in maternal protectiveness was associated with a 13% increase in the odds of children being overweight or obese. The results provide evidence of a relationship between maternal protectiveness and child overweight and obesity, however further research is required to understand the mechanism(s) that links the two concepts.

## Introduction

Overprotective parents are defined as parents who are highly supervising, have difficulties with separation from the child, discourage independent behaviour and are highly controlling [1]. In recent years, the concept of overprotective parenting has received increasing public attention, inspiring a number of colloquial labels including ‘helicopter parenting’, ‘bulldozer parenting’ and more recently, ‘chauffeur parenting’. The interest appears to be motivated by uncertainty about perceived changes in modern parenting practices and concerns about the potential impacts that overprotective parenting might have for children. Some suggested impacts of overprotective parenting include mental health problems, lack of independence and resilience, and increased obesity [2], [3], however these suggested associations remain largely untested.

Though the literature is limited, studies examining the impacts of overprotective parenting on child development have begun to emerge in recent years. The focus of these studies has been on social and emotional outcomes, either for very young children [4], [5] or those entering early adulthood [6], [7]. For example, Cooklin et al. [5] found that higher levels of maternal protectiveness were associated with poorer socio-emotional functioning in 2–3 year old children. At the other end of the developmental spectrum, surveys of college students have found that students who reported having controlling or overprotective parents also reported higher levels of depression and less satisfaction with life [7], as well as reduced self-efficacy [6]. While these studies suggest that overprotective parenting may be associated with poorer social and emotional outcomes for children and young adults, there are still substantial gaps in the literature regarding other stages of child development or developmental outcomes.

The particular parenting behaviours that comprise parental overprotection may vary according to the developmental needs of the child. As such, the implications of overprotective behaviours are also likely to vary for children of different ages. For example, an overprotective parent of a young child might discourage independent activities, which may stifle opportunities for unstructured play and creativity. An overprotective parent of a young adolescent may not allow them to walk or ride to school, impacting on their ability to be independently mobile or active. News stories have also documented overprotective “helicopter” parents of college students attending classes with their children for the first week of college, or intervening with professors if their child receives an unexpectedly low grade [8]. These intrusive behaviours could limit opportunities for young people to learn about risk and taking personal responsibility.

The extent to which parental protectiveness becomes *overprotective* largely depends on the appropriateness of the parenting behaviours given the risks to which children may be exposed [3]. In popular media, overprotective parenting is often positioned as a problem that mainly exists among middle- and upper-class families [9], and while some evidence supports this view [10], other studies show that overprotective parenting is characterised by markers of disadvantage. For example, high maternal separation anxiety has been associated with financial hardship, poor neighbourhood quality and inadequate levels of social support [11]. Maternal overprotection is also more prevalent in families with younger mothers, lower maternal education and fewer children [1], [12]. Concerns about child safety, though common to most families, are also more prevalent in disadvantaged families [13]. These patterns may reflect the different challenges faced by families from lower or higher socio-economic backgrounds, and context is therefore important. Younger children, and children living in high-risk environments such as in neighbourhoods with high levels of crime and violence for example, may be well served by parents who are concerned by their child's young age or the safety of the environment and who act accordingly to protect their child. The extent to which protective parenting is a problem for children can therefore vary from family to family.

There are suggestions that the prevalence of overprotective parenting has increased over time [3] and particularly in the last two decades [10]. One possible reason for this shift is that overprotection increasingly represents normative parenting behaviour because more parents perceive the world as a dangerous place for children. Therefore, heightened vigilance is ‘normal’ and parents who do not conform to the new standards may be considered as ‘bad parents’ [10]. As parental fear becomes normalised, any potential consequences of this parenting style also become normalised, including limitations on the amount of outdoor or free play that children are permitted to engage in. A review of the relevant literature pointing to a decline in levels of outdoor and free play in recent decades supports this view [14]. A study of mothers in the United States, for example, suggested that while 70% of mothers reported they had played outdoors daily as a child, only 31% said their child did the same. The majority of mothers (82%) also reported they restricted outdoor play because of safety concerns [15].

Parental fear is easily understood as a motivator for overprotective parenting, however the events that give rise to the worst parental fears generally have a very low risk of occurring. The likelihood of a child being abducted, murdered or harmed by a stranger is very low [16], and in cases that are reported, an estimated 85% of child sexual assaults are committed by people known to the child, rather than the stereotypical stranger [17]. Additionally, although injuries have been a leading cause of death and hospitalisation for children, both in Australia [18] and worldwide [19], the rates of child deaths and hospitalisations due to preventable injury have generally decreased over time. Between 1986 and 2006 in Australia, the child mortality rate decreased from 30 to 13 deaths per 100,000, a reduction mainly attributable to a decrease in deaths from transport accidents [18]. Other studies have shown that the rate of injury-related hospitalisations for children aged 14 years or less has slowly but consistently declined since the mid-1990′s [20], [21], and that the rate of serious or fatal pedestrian injuries for children decreased by 7.4% each year between 1998 and 2006 [22]. It is possible these declines have occurred as a result of parents becoming more protective, although other changes such as mandatory bicycle helmet laws and improvements to vehicle safety could also explain improvements in child safety.

As perceptions of child safety have become more conservative over time, there have been other apparent generational shifts in levels of physical activity undertaken and the prevalence of overweight and obesity in children and adolescents. Though there is limited research examining trends in overall physical activity levels for Australian children over time, there is some evidence to suggest that the aerobic fitness of Australian children has declined by 4% per decade since 1970 [23], [24]. Other research has shown that among 9–13 year olds the frequency of walking or cycling to or from school and the frequency of physical education classes declined between 1985 and 2001, particularly amongst children from low socio-economic status schools [25]. Other studies have noted a downward trend in the proportion of children walking to school or using other modes of active transport [26]. Though these changes may help explain some of the decrease in child mortality and hospitalisations, the downward trends in physical activity are concurrent with increases in the prevalence of childhood obesity, asthma and allergy, and some mental health problems, all of which have been claimed to be at least partially due to the reduction in time children spend being physically active and being outdoors [27]. Of particular concern, and the main focus of this study, is the increase in childhood obesity. In Australia, the prevalence of child overweight and obesity more than doubled between 1985 and 1996, increasing from 10.2% to 21.6% for boys, and from 11.6% to 24.3% for girls. Between 1996 and 2008 the estimated prevalence plateaued at 23.7% for boys and 24.8% for girls [28]. Though the increase in the prevalence of overweight and obesity appears to have slowed, the prevalence rates are high and remain a serious public health concern.

Beyond the overprotective parenting literature, there is an increasing amount of evidence linking parental fear and the level of physical activity in children. As discussed, parental fear is thought to be a key factor in the decline in the amount of free and outdoor play undertaken by children [14]. Australian research also suggests that a significant number of parents identify ‘stranger danger’ as a barrier to children's independent mobility within their community [29], [30]. Other studies have shown that a fear of stranger danger in parents was significantly associated with parental rules for playing outside, and there was a significant negative association between fear of stranger danger and children's frequency of outdoor physical activity in the neighbourhood [31], though there was no significant association between fear of stranger danger and other physical activity measures, amount of screen time or BMI z-score. Parents who report being concerned about road safety and stranger danger also limit the amount of physical activity and active transport undertaken by children and adolescents [32], [33], and adolescents who spend more time unsupervised after school have been reported to be more physically active than those who spend less time unsupervised [34]. Despite the links between parental fear and children's physical activity, no study as yet has linked overprotection or parental fear with child overweight or obesity outcomes.

If there is a link between parental protectiveness and child BMI, it is more likely to emerge as children become older and more capable of independence. All young children require some level of adult supervision; as such the differences in activity levels between children with overprotective parents and those with average protectiveness may be relatively minor. In contrast, higher levels of parental supervision are not conducive to independent mobility and physical activity in older children [27]. The differences in the activities of older children with overprotective parents and those with average parents will therefore be much wider, for example between the children who are driven to school each day and those who are allowed to walk or ride. Therefore the effects of protectiveness on child BMI would likely become more apparent over time.

To summarise, the evidence suggests that the physical activity of children has declined over time as rates of child overweight and obesity have increased. At the same time, there has been a shift in perceptions of safety for children, even though children arguably face the same or fewer risks today than in previous decades. Parents have become more risk averse and protective over time, and as a result children have enjoyed fewer opportunities for active free play and independent mobility. It is possible therefore, that one of the many factors related to a decline in children's physical activity levels – and perhaps contributing to the increase in obesity – is the increase in parental fear and protectiveness.

For this study, we aimed to determine if there was an association between levels of parental protectiveness and child body mass index (BMI), and if so, the nature of that relationship over time. We also aimed to examine the demographic characteristics of highly protective parents and to provide clarity to the questions regarding the typical characteristics of highly protective parents. We used data from *Growing Up in Australia: The Longitudinal Study of Australian Children* (LSAC) to examine the study aims. The LSAC followed the same children every two years from the age of 4–5 years to 10–11 years, allowing us to examine how the relationship between protective parenting and child overweight and obesity develops over time. For some families, higher levels of protectiveness may be an appropriate response to the environment in which they live. We therefore refer to parents as being highly protective, rather than overprotective, to avoid the implication that such a parenting style is undesirable for all families.

## Methods

### Study Design and Population

The LSAC is a nationally representative and multi-disciplinary study of Australian children and their families. Commencing in 2004, data were collected every two years from two cohorts of children; 5107 infants aged 3–19 months (B-cohort) and 4983 children aged 4 years 3 months to 5 years 7 months (K-cohort). In order to follow children through a period of development where highly protective parenting may have greater impact on the development of obesity we focussed on the K-cohort for this study. The K-cohort children were revisited in 2006 (Wave 2, age 6–7 years), 2008 (Wave 3, age 8–9 years) and 2010 (Wave 4, age 10–11 years). Of the 4983 families that participated at Wave 1, 4464 (89.6%) participated at Wave 2, 4332 (86.9%) at Wave 3 and 4164 (83.6%) at Wave 4.

The LSAC employed a two-stage clustered sample design, with Australian postcode area as the primary sampling unit, and the sampling frame drawn from the Medicare Australia enrolment database. Approximately one in ten Australian postcode areas were randomly selected and children were then randomly selected within postcode areas ensuring that only one child per household was selected (the study child). The initial response rate at Wave 1 was 47% for the K-cohort, with the initial sample broadly representative of the Australian population of families with children in the LSAC age groups when compared with 2001 Census data, but slightly under-representative of families who were single-parent, non-English speaking, living in rental properties or living in remote areas [35]. These same characteristics were also over-represented in the sample that dropped out in subsequent waves of the study [36]. Sample and longitudinal weights were developed for the study to adjust for the initial response bias and differential likelihood of ongoing participation [36]. The weights were used in all analyses conducted for the current study.

Data collection methods included parent face-to-face interviews in the home, self-complete parent questionnaires, interviewer observations, direct measures of physical attributes and cognitive development, time-use diaries and mailed out questionnaires for childcare providers and teachers. The person who was interviewed as the primary parent (Parent 1) was the parent who had the most contact with the child, most typically the biological mother (approximately 97% across waves). Questionnaires were also offered to Parent 2, where there was another carer living with the study child (approximately 85% of families). Parent 2 was most typically the biological father of the study child (88–94% across waves).

### Ethics Statement

The LSAC is conducted in a partnership between the Department of Social Services (DSS), the Australian Institute of Family Studies (AIFS) and the Australian Bureau of Statistics (ABS). The study has ethics approval from the Australian Institute of Family Studies Ethics Committee. The Ethics Committee is registered with the Australian Health Ethics Committee, a subcommittee of the National Health and Medical Research Council (NHMRC). As the study children were all minors at the time these data were collected, written informed consent was obtained from the caregiver on behalf of each of the study children. The signed consent forms are retained by the field agency (ABS). Individual and organisational licenses to access the confidentialised data sets are available upon application to the DSS [37].

### Maternal Protectiveness

The parental protectiveness measures were collected in the Parent 1 and Parent 2 Questionnaires at Wave 2 (6–7 years) and Wave 3 (8–9 years). Due to lower response rates from fathers on the Parent 2 Questionnaire at Waves 2 (78%) and 3 (72%), and to ensure that no additional response bias would be introduced through the exclusion of lone-parent families, we restricted analysis to respondents who were mothers of the study child. Including data from fathers would reduce the analytic sample by approximately 30%.

The protective parenting measure consisted of 3 items that were selected for the LSAC from a larger, validated 8-item overprotective parenting scale [4]. The items were; “How often do you try to protect this child from life's difficulties?”, “How often do you put this child's wants and needs before your own?” and “How often does leaving this child with other people upset you no matter how well you know them?” Parents could respond 1  =  never/almost never; 2  =  rarely; 3  =  sometimes; 4  =  often or 5  =  almost always/always. Scores were summed to produce a total protectiveness score at Wave 2 (6–7 years) and Wave 3 (8–9 years). Scores could range from 3 to 15, with higher scores reflecting higher levels of protectiveness. The distribution of each total score was close to a normal distribution with only a slight negative skew. To facilitate interpretation, total scores were standardised to have a mean of 0 and standard deviation of 1. The internal consistency of responses to the three items at Wave 2 and Wave 3 was low (Wave 2 α  =  0.57, Wave 3 α  =  0.57), though Cronbach's alpha can be an inaccurate measure of reliability when the number of items is small [38]. The between-item correlations ranged from *r*  =  0.24 to *r*  =  0.41 at Wave 2 and from *r*  =  0.26 to *r*  =  0.40 at Wave 3 (see Table 1). The correlation between the total summed scores at Wave 2 (6–7 years) and Wave 3 (8–9 years) was *r*  =  0.55, indicating that maternal protectiveness scores were reasonably consistent between Wave 2 (6–7 years) and 3 (8–9 years), given the measures were collected approximately two years apart. A very strong correlation between waves is not necessarily expected because the construct could reasonably change over time as family circumstances change, for example as families expand or as mothers increase their participation in the workforce.

**Table 1:** Correlation matrix of scale items and total scores on the protective parenting scale at Wave 2 (6–7 years) and Wave 3 (8–9 years).

	Wave 2 Protectiveness				Wave 3 Protectiveness			
	(6–7 years)				(8–9 years)			
	Protect	Wants and Needs	Leaving Child	Total Wave 2 Score	Protect	Wants and Needs	Leaving Child	Total Wave 3 Score
**Wave 2 Protectiveness**								
**(6**–**7 years)**								
Protect	1.00							
Wants and needs	.41	1.00						
Leaving child	.26	.24	1.00					
Total Wave 2 Score	.75	.68	.75	1.00				
**Wave 3 Protectiveness**								
**(8**–**9 years)**								
Protect	.43	.26	.24	.42	1.00			
Wants and needs	.24	.43	.20	.37	.40	1.00		
Leaving child	.17	.17	.53	.42	.27	.26	1.00	
Total Wave 2 Score	.37	.37	.46	.55	.73	.69	.76	1.00

Protect  =  How often do you try to protect this child from life's difficulties?

Wants and needs  =  How often do you put this child's wants and needs before your own?

Leaving child  =  How often does leaving this child with other people upset you no matter how well you know them?

All correlation values are significant at p < .05.

The properties of the protective parenting measure used in the LSAC were also assessed in another study using data from the B-cohort to examine the extent to which maternal separation anxiety, overprotective parenting and maternal mental health were related or separate constructs [5]. In that study, maternal separation anxiety (at age 3–19 months) had a low, but statistically significant correlation with total maternal protection scores when children were 2–3 years of age (r  =  0.32). Maternal protectiveness also had a low and significant correlation with maternal mental health (r  =  0.14). Cooklin et al. [5] concluded that although the 3-item scale has limited sensitivity and less than optimal internal consistency, the items adequately assessed the construct and had discriminant validity from separation anxiety and maternal mental health.

### Child BMI, overweight and obesity

BMI is a widely accepted measure for identifying children and adolescents with excess weight [39]. Child BMI was calculated as weight in kilograms divided by the squared height in metres (weight/height^2^). Measures of child height and weight were collected at each wave by the interviewers. Children were weighed in light clothing to the nearest 50g using glass bathroom scales provided by the interviewer. Two height recordings were collected, without shoes, using a portable rigid stadiometer. The average of the two height measurements was used for analyses. Where the two measurements differed by more than 0.5 centimetres, a third measurement was taken and the average of the two closest measures was used. International Obesity Taskforce (IOTF) age- and sex-specific BMI cut-points [40] were used to determine if children fell into the normal, overweight or obese BMI range at each wave.

### Maternal and family characteristics

The variables described below were assessed to determine their association with maternal protectiveness, and were also included as potential covariates that relate to child BMI. Most of the covariates were repeatedly collected at each wave, however variables that largely remained stable over time were taken from Wave 1 (4–5 years) measures to coincide with the intercept of the longitudinal model. Less stable factors, such as maternal employment or mental health status, were aggregated over time and are described in further detail below.

#### Measures from Wave 1 (4–5 years)

Covariates included *maternal BMI*, calculated from self-reports of height and weight (up to 25 or normal, 25 up to 30 or overweight, and 30 plus, or obese), *mother's age at the birth of her first child* (up to 19 years, 20–24 years, 25–29 years and 30 years or older) and *mother's highest educational attainment* (less than Year 12, Year 12, post-school qualification). *Household income* was included as a measure of family wealth. At Wave 1, family income was collected in broad categories, and are grouped here as up to $599 per week, $600–$999 per week, $1000–$1999 per week and $2000 per week or more. *Socio-economic index for areas* (SEIFA) was used to examine the effects of relative neighbourhood disadvantage. SEIFA is a summary measure of the socio-economic conditions of people living in an area, derived by the Australian Bureau of Statistics. It is scored on a continuum of disadvantage (low values) to advantage (high values) which is derived from census variables related to both advantage and disadvantage such as income and tertiary education. SEIFA values were divided into tertiles at each wave (low, middle and high).

#### Measures collated over time

*Maternal employment pattern* was a derived variable summarising the degree of employment over the four waves. Employment status was collected at each wave and given a score. Mothers who were not working (either unemployed or not in the labour force) received a score of 0. Mothers on maternity leave received a score of 1, those in part-time work (up to 30 hours per week) a score of 2, and those with full-time employment (more than 30 hours per week) a score of 3. These scores were summed across all four waves, resulting in a total score that could range from 0 (never employed) to 12 (always full-time). Mothers were then broadly grouped according to their total score; not working (1 point or less); some part-time employment (2–5 points); consistent part-time (6–8 points) or mostly full-time (9 points or more).

To address any concerns that the measure of maternal protectiveness may reflect an underlying anxiety disorder, a measure of *maternal mental health* was included. This was measured using the Kessler K6 scale [41], a commonly used 6-item assessment of psychological distress and anxiety disorder. Scores on the scale can range from 0 to 24, with higher scores representing poorer psychological functioning. In line with other studies [42], [43] this study used a cut-off of 8 to signal likely psychological distress. The proportion of mothers with likely psychological distress was 16% at Wave 1 (4–5 years), 11% at Wave 2 (6–7 years), 14% at Wave 3 (8–9 years) and 13% at Wave 4 (10–11 years). The measure was collated across waves to provide a summary indication of mental health, in terms of the number of waves mothers had likely psychological distress; 0, 1–2 or 3–4 waves. Finally, the *sibling position* of the study child was also examined, and taken as at Wave 4 (10–11 years), to account for the introduction of new siblings over time. The categories included none (only child), youngest child, middle child and eldest child.

### Data Analysis

SAS 9.3 was used to conduct all analyses. Basic descriptive analyses were used to examine how standardised maternal protectiveness scores varied according to family and demographic characteristics. Multivariate regression models were also fitted to determine the characteristics that were independently associated with higher maternal protectiveness scores. A generalised estimating equations (GEE) model, often used for repeated measures data as it accounts for the within-subject correlation between observations across time and can allow for missing data in the outcome measure [44], was used for the longitudinal model. An unstructured working correlation matrix was specified for the model. Because of the relatively large sample size and the small number of covariance parameters, an unstructured correlation matrix does not adversely impact the power of the study compared with a more prescriptive covariance structure. Child age group (or wave) was used as the key predictor representing time. The longitudinal model estimated the odds of the study child being overweight or obese at Wave 1 (4–5 years), and the change in these odds at Wave 2 (6–7 years), Wave 3 (8–9 years) and Wave 4 (10–11 years), according to the predictor variable set. These change estimates were then used to determine the overall odds of child overweight and obesity at each wave for each predictor in the model. Covariates that were significantly associated with the odds of overweight or obesity, either at Wave 1, or in the change in odds at subsequent waves were retained in the final model. Variables that were not significantly associated with child overweight and obesity were excluded so that the most parsimonious model was achieved.

With attrition across waves and missing data on the Parent 1 questionnaire at Wave 2 (6–7 years) and 3 (8–9 years) where the protective parenting measure was collected, the final analytic sample consisted of 2,933 families. All analyses were weighted using the longitudinal study weights provided with the LSAC data set.

## Results

### Maternal protectiveness and demographic characteristics

Table 2 provides the Wave 2 (6–7 years) and Wave 3 (8–9 years) unadjusted mean protectiveness scores by demographic characteristics, along with estimates from the multivariate linear regression analyses. There was a clear socio-economic gradient, with higher standardised protectiveness scores observed for mothers with greater levels of disadvantage. For example, the mean standardised protectiveness score when children were 6–7 years (Wave 2) was 0.16 for mothers in the lowest income category, and −0.34 for those in the highest income category. This difference translates to a gap of 0.5 of a standard deviation in protectiveness scores for families in the highest and lowest income categories, a moderate effect size. In the adjusted multivariate regression models, the difference was 0.18 (*p*  =  .023) after accounting for maternal education, employment, age at first child, mental health and neighbourhood disadvantage. Significantly higher standardised protectiveness scores were also found for mothers with lower levels of education, younger first-time mothers, mothers with mental health difficulties at multiple waves, mothers who were not working, and those living in neighbourhoods with greater disadvantage.

**Table 2:** Mean standardised maternal protectiveness scores at Wave 2 (6–7 years) and Wave 3 (8–9 years) by demographic characteristics, and adjusted multivariate regression coefficients.

	Wave 2 (6–7 Years)				Wave 3 (8–9 Years)			
**Factor**	N	Mean Score (SE)	Regression Coefficient	p-value	N	Mean Score (SE)	Regression Coefficient	p-value
**Mother's highest education**								
Post-school	1575	−0.20 (0.03)	Ref		1660	−0.22 (0.03)	Ref	–
Year 12	459	−0.03 (0.05)	0.08	.192	496	0.02 (0.05)	0.15	.012
Less than Year 12	1130	0.17 (0.04)	0.22	<.001	1254	0.13 (0.03)	0.23	<.001
**Maternal employment pattern**								
Not working	345	0.28 (0.06)	Ref		387	0.19 (0.06)	Ref	–
Some part-time	593	−0.01 (0.05)	−0.22	.008	647	0.02 (0.05)	−0.13	.120
Mostly part-time	1059	−0.05 (0.03)	−0.19	.008	1129	−0.05 (0.03)	−0.15	.039
Mostly full-time	1144	−0.09 (0.03)	−0.22	.002	1234	−0.08 (0.03)	−0.16	.012
**Mothers age at first child**								
30 years or more	1131	−0.13 (0.03)	Ref		1162	−0.10 (0.03)	Ref	–
25–29 years	1297	−0.05 (0.03)	0.02	.677	1376	−0.06 (0.03)	0.00	.960
20–24 years	578	0.17 (0.04)	0.18	.003	668	0.08 (0.04)	0.08	.188
Less than 19 years	155	0.34 (0.10)	0.23	.023	199	0.24 (0.08)	0.13	.155
**No. of waves maternal mental health problem**								
None	2358	−0.06 (0.03)	Ref		2524	−0.11 (0.02)	Ref	–
One-Two	675	0.14 (0.05)	0.13	.010	736	0.17 (0.04)	0.22	<.001
Three-Four	138	0.14 (0.09)	0.16	.105	163	0.32 (0.10)	0.38	<.001
**Household income**								
$2000 or more per week	422	−0.34 0.05)	Ref		447	−0.32 (0.04)	Ref	–
$1000–$1999 per week	729	−0.09 (0.04)	0.15	.018	772	−0.10 (0.04)	0.12	.038
$600–$999 per week	1348	0.04 (0.03)	0.19	.003	1464	0.04 (0.03)	0.18	.001
Up to $599 per week	495	0.16 (0.06)	0.18	.023	548	0.11 (0.05)	0.14	.046
**Neighborhood disadvantage (SEIFA)**								
Least disadvantaged tertile	1027	0.16 (0.04)	Ref		945	−0.21 (0.04)	Ref	–
Middle tertile	1235	−0.01 (0.04)	0.08	.147	1336	−0.01 (0.03)	0.09	.056
Most disadvantaged tertile	909	−0.20 (0.04)	0.15	.017	1142	0.12 (0.04)	0.14	.016

Included in initial models but removed due to non-significance: Maternal BMI, family structure, position of child amongst siblings and child gender.

### Maternal protectiveness and child overweight and obesity

The proportions of children in the normal, overweight and obese BMI categories at each wave are provided in Table 3. The proportion of overweight children increased from 15% at Wave 1 (4–5 years) to 20% at Wave 4 (10–11 years). Around 5–6% of children were obese at each wave, and approximately 65% of study children were always in the normal BMI range. Only 1.5% of children were obese at all four waves (not shown). Table 3 also shows that children who were overweight or obese children at any given wave had significantly higher Wave 2 (6–7 years) and Wave 3 (8–9 years) maternal protectiveness scores than children of normal weight.

**Table 3:** Proportion of children categorised as being normal weight, overweight or obese at each wave, with mean Wave 2 (6–7 years) and Wave 3 (8–9 years) standardised maternal protectiveness scores.

		Wave 2 (6–7 Years)		Wave 3 (8–9 Years)	
	N (weighted %)	Mean Score (SE)	p-value	Mean Score (SE)	p-value
**Wave 1 (4**–**5 years)**					
Normal	2431 (79.6)	−0.03 (0.02)	ref	−0.08 (0.03)	ref
Overweight	461 (15.4)	0.07 (0.05)	.350	−0.06 (0.05)	.777
Obese	147 (5.0)	0.15 (0.09)	.034	0.19 (0.09)	.005
					
**Wave 2 (6**–**7 years)**					
Normal	2499 (81.5)	−0.03 (0.02)	ref	−0.08 (0.03)	ref
Overweight	392 (13.1)	0.06 (0.06)	.145	−0.06 (0.06)	.730
Obese	148 (5.4)	0.20 (0.09)	.019	0.30 (0.09)	<.001
					
**Wave 3 (8**–**9 years)**					
Normal	2368 (76.7)	−0.05 (0.02)	ref	−0.09 (0.03)	ref
Overweight	497 (16.8)	0.07 (0.05)	.028	−0.03 (0.05)	.281
Obese	174 (6.5)	0.26 (0.08)	<.001	0.31 (0.08)	<.001
					
**Wave 4 (10**–**11 years)**					
Normal	2132 (73.6)	−0.07 (0.03)	ref	−0.10 (0.03)	ref
Overweight	555 (20.1)	0.04 (0.05)	.036	−0.06 (0.05)	.421
Obese	156 (6.3)	0.29 (0.09)	<.001	0.34 (0.08)	<.001

All of the covariates examined in Table 2 were included in the initial longitudinal model. Covariates with a p-value greater than *p*  =  .10 for both Wave 1 (4–5 years) estimates and change estimates at later waves were removed until the most parsimonious model was achieved. Including both the Wave 2 (6–7 years) and Wave 3 (8–9 years) measures of maternal protectiveness in the model resulted in unstable estimates for both predictors. When entered into the model separately, the Wave 3 measure of maternal protectiveness was a statistically significant predictor of child overweight and obesity but the Wave 2 measure was not. The Wave 2 measure was therefore excluded from further analysis, and all further references to the effects of maternal protectiveness refer to the measure collected at Wave 3 (8–9 years).

The longitudinal GEE model results estimating the odds ratios of child overweight and obesity at each wave are provided in Table 4, and the extent to which those odds ratios changed at each wave are provided in Table 5. There was no significant association between maternal protectiveness and child overweight and obesity at age 4–5, 6–7 or 8–9 years. At 10–11 years, a 1 standard deviation increase in maternal protectiveness was associated with a 13% increase (OR  =  1.13, *p*  =  .018) in the likelihood of the child being overweight or obese. An increase of 2 standard deviations, for example those 1 standard deviation above the mean compared to 1 standard deviation below the mean, was associated with a 29% increase (OR  =  1.29, *p*  =  .018) in the odds of children being overweight or obese. These results were observed after controlling for maternal BMI, household income, maternal mental health and sibling position.

**Table 4:** Estimated odds of children being overweight or obese at each wave, according to maternal protectiveness and other family characteristics.

	Wave 1 (4–5 Years)			Wave 2 (6–7 Years)			Wave 3 (8–9 Years)			Wave 4 (10–11 Years)		
	OR	95% CI	p	OR	95% CI	p	OR	95% CI	p	OR	95% CI	p
**Maternal protectiveness**												
At age 8–9 Years + 1 SD	0.97	(0.87, 1.08)	.578	1.02	(0.91, 1.14)	.758	1.07	(0.96, 1.19)	.203	1.13	(1.02, 1.26)	.018
At age 8–9 years +2 SD	0.94	(0.75, 1.17)	.578	1.04	(0.82, 1.31)	.758	1.15	(0.93, 1.42)	.203	1.29	(1.04, 1.58)	.018
**Maternal BMI (Wave 1)**												
Normal	Ref			Ref			Ref			Ref		
Overweight	1.82	(1.42, 2.31)	<.001	1.96	(1.51, 2.55)	<.001	2.19	(1.83, 2.78)	<.001	2.37	(1.89, 2.98)	<.001
Obese	2.19	(1.66, 2.88)	<.001	2.99	(2.24, 3.98)	<.001	2.81	(2.15, 3.68)	<.001	3.38	(2.62, 4.38)	<.001
**Household Income (Wave 1)**												
$2000 or more per week	Ref			Ref			Ref			Ref		
$1000–$1999 per week	1.05	(0.74, 1.49)	.795	1.25	(0.84, 1.87)	.276	1.02	(0.72, 1.45)	.899	1.21	(0.86, 1.71)	.281
$600–$999 per week	1.03	(0.74, 1.42)	.869	1.29	(0.89, 1.86)	.184	1.05	(0.76, 1.44)	.786	1.40	(1.02, 1.92)	.039
Up to $599 per week	1.47	(1.00, 2.15)	.047	2.06	(1.34, 3.15)	<.001	1.91	(1.32, 2.78)	<.001	2.08	(1.42, 3.04)	<.001
**No. of waves maternal mental health problem**												
None	Ref			Ref			Ref			Ref		
One-two	0.89	0.68, 1.16	.384	0.85	0.64, 1.13	.261	1.21	0.94, 1.55	.133	1.21	0.95, 1.53	.125
Three-four	0.92	0.56, 1.49	.723	0.40	0.21, 0.73	.003	0.52	0.32, 0.87	.013	0.48	0.29, 0.78	.003
**Position among siblings**												
Only child												
Youngest child	0.77	0.53, 1.12	.172	0.65	0.44, 0.94	.023	0.74	0.52, 1.04	.086	0.85	0.61, 1.18	.328
Middle child	0.72	0.47, 1.10	.131	0.57	0.37, 0.88	.012	0.51	0.33, 0.77	.001	0.48	0.33, 0.72	.003
Eldest child	0.84	0.57, 1.22	.349	0.65	0.44, 0.95	.027	0.65	0.45, 0.93	.018	0.59	0.42, 0.83	.003

OR  =  Odds ratio; CI  =  Confidence Interval. Included in initial models but removed due to non-significance: Family structure, maternal employment pattern, maternal age at birth of first child and neighbourhood disadvantage. Maternal protectiveness at Wave 2 (6–7 years) removed due to collinearity.

**Table 5:** Estimated change in the odds of children being overweight or obese at Wave 2 (6–7 years), Wave 3 (8–9 years) and Wave 4 (10–11 years) according to maternal protectiveness and other family characteristics, relative to Wave 1 (age 4–5 years).

	Interaction Terms								
	Wave 2 (6–7 Years)			Wave 3 (8–9 Years)			Wave 4 (10–11 Years)		
	OR	95% CI	*p*	OR	95% CI	*p*	OR	95% CI	*p*
**Reference Group (intercept)**	0.77	(0.52, 1.12)	.173	1.11	(0.74, 1.66)	.631	1.06	(0.68, 1.64)	.811
**Maternal protectiveness**									
At age 8–9 Years	1.05	(0.95, 1.16)	.322	1.11	(1.00, 1.22)	.049	1.17	(1.04, 1.31)	.007
**Maternal BMI (Wave 1)**									
Normal	Ref			Ref			Ref		
Overweight	1.08	(0.87, 1.34)	.475	1.21	(0.96, 1.52)	.111	1.31	(1.02, 1.67)	.033
Obese	1.36	(1.09, 1.71)	.007	1.29	(1.01, 1.64)	.045	1.55	(1.18, 2.03)	.002
**Household Income (Wave 1)**									
$2000 or more per week	Ref			Ref			Ref		
$1000–$1999 per week	1.19	(0.87, 1.63)	.270	0.98	(0.71, 1.35)	.884	1.15	(0.81, 1.66)	.433
$600–$999 per week	1.25	(0.92, 1.70)	.151	1.02	(0.75, 1.38)	.915	1.36	(0.97, 1.90)	.074
Up to $599 per week	1.40	(0.98, 2.00)	.064	1.30	(0.90, 1.88)	.157	1.42	(0.94, 2.13)	.098
**No. of waves maternal mental health problem**									
None	Ref			Ref			Ref		
One-two	0.96	(0.77, 1.19)	.687	1.36	(1.06, 1.75)	.016	1.36	(1.04, 1.77)	.025
Three-four	0.43	(0.25, 0.75)	.003	0.57	(0.36, 0.92)	.022	0.52	(0.30, 0.91)	.021
**Position among siblings**									
Only child	Ref			Ref			Ref		
Youngest child	0.84	(0.61, 1.16)	.288	0.96	(0.67, 1.37)	.806	1.10	(0.75, 1.61)	.622
Middle child	0.80	(0.56, 1.12)	.195	0.71	(0.48, 1.05)	.082	0.67	(0.44, 1.03)	.070
Eldest child	0.78	(0.56, 1.08)	.128	0.78	(0.54, 1.11)	.164	0.71	(0.49, 1.03)	.073

OR  =  Odds ratio; CI  =  Confidence Interval. Included in initial models but removed due to non-significance: Family structure, maternal employment pattern, maternal age at birth of first child and neighbourhood disadvantage. Maternal protectiveness at Wave 2 (6–7 years) removed due to collinearity.

The strongest predictor of child overweight and obesity was maternal BMI. Children were more than twice as likely (OR  =  2.19, *p* < .001) to be overweight or obese at age 4–5 years if their mother was obese compared to children of mothers in the normal weight range. These odds ratios increased to 2.99 (*p* < .001) at age 6–7 years, 2.81 (*p* < .001) at age 8–9 years and 3.38 (*p* < .001) at age 10–11 years. The odds ratios were significantly higher at Waves 2, 3 and 4 (see Table 5), indicating that the effect of maternal BMI significantly increased over time.

Household income was also associated with the odds of child overweight and obesity. Children in the lowest category of household income (up to $599 per week) for example, were nearly 1.5 times more likely (OR  =  1.47, *p*  =  .047) than children in the highest income category ($2000 or more per week) to be overweight or obese at age 4–5 years. At subsequent waves, children in the lowest income category were approximately twice as likely to be overweight or obese as children in the highest income category (e.g. 10–11 years OR  =  2.08, *p* < .001). These effects did not significantly increase over and above the effect observed for Wave 1 (4–5 years, see Table 5).

The effects of maternal mental health depended on the persistence of problems across time. Children of mothers who had likely psychological distress at one or two waves were no more or less likely to be overweight or obese at any wave than children whose mother never had likely psychological distress. Children of mothers with likely psychological distress at three or four waves had significantly lower odds of child overweight or obesity at age 6–7 years (OR  =  0.40, *p*  =  .003), 8–9 years (OR  =  0.52, *p*  =  .013) and 10–11 years (OR  =  0.48, *p*  =  .003, see Table 4).

Finally, the study child's position amongst their siblings was also associated with the likelihood of overweight and obesity. Compared to being an only child, children with siblings were significantly less likely to be overweight or obese, and for children who had younger siblings in particular. Study children who were the middle child, for example, were about half as likely (OR  =  0.48, *p*  =  .003) to be overweight or obese at age 10–11 years than children without siblings.

## Discussion

The literature on overprotective parenting and the potential impacts on child outcomes has been gaining momentum in recent years. As parents increasingly perceive the world to be a dangerous place for children [45], parenting styles have adapted and become more protective over time [3], [27]. At the same time, other changes appear to have occurred, including a decline in physical activity [23], [24], [25], [26], [27], a decline in the amount of outdoor play [14], [15] and an increase in the prevalence of childhood obesity [28]. Though these patterns might suggest that higher levels of protective parenting could be linked to child BMI, there is no direct evidence to support this hypothesis and certainly none that examines the relationship from a longitudinal perspective. There has also been conflicting evidence regarding the family and demographic characteristics that are associated with a highly protective parenting style [1], [10], [11]. In this study, we aimed to determine if any link could be drawn between protective parenting and child overweight and obesity, and if so the nature of that relationship over time. We also aimed to address the uncertainty regarding the social and demographic correlates of maternal protectiveness.

There are two noteworthy features of our study. The first was our finding that higher maternal protectiveness was associated with an increased likelihood of child overweight and obesity over time, after the effects of household income, maternal BMI, maternal mental health, maternal education and position amongst siblings were controlled for. Though there has been speculation about this connection [2], this is the first study to our knowledge to provide evidence of this link. Maternal protectiveness was not significantly associated with child overweight and obesity at younger ages, however at 10–11 years of age a one standard deviation increase in maternal protection scores was associated with a 13% increased likelihood of child overweight and obesity. Though this may be considered a small or negligible effect, more than a quarter of 10–11 year old children were overweight or obese in our study, which is on par with population-level estimates [28]. A small increase in the likelihood of overweight and obesity may therefore be relevant for a large number of children, and particularly as highly protective parenting becomes the norm for an increasing number of families.

The use of longitudinal data, a particular strength of this study, was also important in identifying how this relationship emerged over time. Maternal protectiveness was not significantly associated with child overweight and obesity until children were 10–11 years of age. While it is possible that this pattern of results was due to the timing of the protectiveness measure, collected when children were aged 8–9 years, the finding supports our hypothesis that any effects of maternal protectiveness would not emerge until a stage where children can reasonably be expected to become more independent of their parents. This is a particularly interesting finding in the context of other research suggesting that a large component of childhood obesity is established by the age of 5, where children who were overweight at age 5 were four times as likely as normal-weight children to become obese by age 14 [46]. Our pattern of results contributes to previous research by suggesting that while there are many factors that contribute to obesity trajectories from an early age, there are other factors that influence those trajectories throughout childhood beyond early developmental periods. Protective parenting styles, which could be characterised by a reluctance to allow children to be independently mobile and therefore less active, provides a potential explanation for later emergence of overweight and obesity.

Our results for maternal protectiveness were adjusted for maternal mental health. This approach was taken to address concerns that the protectiveness measure may have reflected general anxiety rather than protectiveness, particularly as one of the items related to parents becoming upset at leaving the child with other people irrespective of how well they knew them. In addition, we found that mothers with mental health difficulties also had higher protectiveness scores on average than mothers without such difficulties. Despite these similarities, the results of the longitudinal model showed that there was no significant difference in the odds of child overweight or obesity for mothers who had a likely psychological distress at one or two waves of the study compared to mothers who did not have psychological distress at any wave. Furthermore, for mothers enduring likely psychological distress at 3 or 4 waves, their children had lower odds of being overweight or obese relative to children whose mother did not have a mental health problem at any wave. As the results for maternal mental health were opposite to those for maternal protectiveness, there is little doubt that the results for maternal protectiveness are independent of any underlying mental health problem among mothers.

Our second key finding was that maternal protectiveness was higher on average among more disadvantaged families, including those with lower household incomes, lower maternal education, living in disadvantaged neighbourhoods, where mothers were not employed and where mothers have mental health issues. Our findings support the scant literature and continue to point to the discrepancy with what is more widely believed about highly protective parents – namely that it is a feature of advantaged families [10]. The ‘typical’ highly protective parent is more likely to be found in disadvantaged circumstances [1], [12].The relationship between socioeconomic disadvantage and parental protectiveness is perhaps not unexpected, current literature notwithstanding, given the potential circumstances and environments that less advantaged families may experience on a daily basis. Families with lower levels of education and income, for example, may have fewer housing options and therefore live in neighbourhoods that are less safe or in areas that contain busy streets and highways. In such contexts parental anxiety about child safety may be more justified.

The relationship with maternal employment pattern, where maternal protectiveness was lower on average for mothers in the workforce could have several underlying explanations. One is that mothers with lower feelings of protectiveness are more likely to return to the workforce after having children because they feel more secure leaving their child in the care of others. In contrast, mothers who have a financial need to return to work may need to suppress feelings of anxiety in order to cope with separation.

There are limitations to the study. Though it is possible that higher maternal protectiveness leads to poorer weight outcomes through limited physical activity or independent mobility for older children, the mechanisms that link high protection and child BMI, and the direction in which these mechanisms operate, remain unclear. While some research certainly points to evidence that suggests that children of highly protective parents are less active [47], such a link is not informed by the data in this study. As such, alternative explanations are also possible. For example, highly protective parents may be more likely to indulge their child's preferences for sweets and junk food, which could potentially explain the link between protectiveness and child overweight and obesity. As with most observational studies, we cannot make conclusions regarding causal relationships, and further research is needed to understand these relationships in more detail. Establishing the mechanism(s) may be a difficult task, however, given the lack of clarity surrounding the biological mechanisms that underpin weight loss [48].

Another limitation relates to the measure used to assess protectiveness. The protective parenting scale adopted for the LSAC has only had limited assessments of reliability or validity. It is therefore possible that the items measure a construct other than maternal protectiveness. Though the scale only had limited items and the internal consistency was unclear, the scale showed good consistency two years apart. We also addressed potential mental health and anxiety problems in mothers by including a maternal mental health measure in our models. Furthermore, the LSAC is an omnibus survey that restricts the inclusion of a broader scale assessing the highly protective parenting construct in greater detail. The advantage of this approach however, is the availability of a wide range of contextual information that may help to understand the emergence of highly protective parenting.

Finally, as this was a longitudinal study the effects of attrition and response bias on the results need to be considered. With each subsequent wave of data collection, the sample became less representative of lone-parent families, non-English speaking families, those living in rental properties or in remote areas. We attempted to correct for these biases by weighting all analyses, with greater weight being provided to the children and families that were less likely to participate at all four waves of the study, and note that the inclusion of study weights did not substantially alter the results of the study.

There are several avenues of further research that will help to shed more light on the impacts of highly protective parenting. Further research is needed to understand the mechanisms through which maternal protectiveness is related to child obesity. One potential avenue for this research is the examination of data collected in the LSAC time-use diaries. These diaries do not provide information on the level of physical activity undertaken by children, but they do provide some information on the types of activities undertaken by children, along with whether these activities were outdoors and if an adult was nearby. Other avenues of research may examine additional factors that are associated with overweight and obesity, such as diet, and the extent to which the diets of children vary for different levels of protectiveness. Finally, this study was restricted to examining maternal protectiveness. Fathers also play a critical role in child development [49], and research indicates that fathers are increasingly spending more time caring for their children [50] and are typically more oriented towards play and activities that encourage risk-taking than mothers [51]. Therefore it is important to extend this research to examine the impacts of paternal protectiveness and how this interacts with maternal protectiveness. For example, how do child development trajectories vary when fathers, or both parents, exhibit highly protective parenting styles?

Our findings show that high maternal protection is linked to increasingly higher odds of child overweight and obesity, and this relationship is independent of other family characteristics that are associated with both maternal overprotection and child BMI. However, as maternal protectiveness was also more prevalent in disadvantaged families, any current public health initiatives that aim to increase children's physical activity need to consider the safety of children living in less advantaged areas, and that parents may well have legitimate safety concerns for their children.
